# A sporadic case of congenital aniridia caused by pericentric inversion inv(11)(p13q14) associated with a 977 kb deletion in the 11p13 region

**DOI:** 10.1186/s12920-020-00790-1

**Published:** 2020-09-18

**Authors:** Tatyana A. Vasilyeva, Andrey V. Marakhonov, Marina E. Minzhenkova, Zhanna G. Markova, Nika V. Petrova, Natella V. Sukhanova, Philipp A. Koshkin, Denis V. Pyankov, Ilya V. Kanivets, Sergey A. Korostelev, Irina A. Krynskaya, Nadezhda V. Shilova, Sergey I. Kutsev, Vitaly V. Kadyshev, Rena A. Zinchenko

**Affiliations:** 1 Research Centre for Medical Genetics, Moscow, Russian Federation; 2 Central Clinical Hospital of the Russian Academy of Sciences, Moscow, Russian Federation; 3 Genomed Ltd., Moscow, Russian Federation; 4 I.M. Sechenov First Moscow State Medical University, Moscow, Russian Federation; 5 N.A. Semashko National Research Institute of Public Health, Moscow, Russian Federation

**Keywords:** Aniridia, balanced chromosomal rearrangements, Local disbalance at the breakpoints, Case report

## Abstract

**Background:**

Because of the significant occurrence of “WAGR-region” deletions among de novo mutations detected in congenital aniridia, DNA diagnosis is critical for all sporadic cases of aniridia due to its help in making an early diagnosis of WAGR syndrome. Standard cytogenetic karyotype study is a necessary step of molecular diagnostics in patients with deletions and in the patients’ parents as it reveals complex chromosomal rearrangements and the risk of having another affected child, as well as to provide prenatal and/or preimplantation diagnostics.

**Case presentation:**

DNA samples were obtained from the proband (a 2-year-old boy) and his two healthy parents. Molecular analysis revealed a 977.065 kb deletion that removed loci of the *ELP4*, *PAX6*, and *RCN1* genes but did not affect the coding sequence of the *WT1* gene. The deletion occurred de novo on the paternal allele. The patient had normal karyotype 46,XY and a de novo pericentric inversion of chromosome 11, inv(11)(p13q14).

**Conclusions:**

We confirmed the diagnosis of congenital aniridia at the molecular level. For the patient, the risk of developing Wilms’ tumor is similar to that in the general population. The recurrence risk for sibs in the family is low, but considering the possibility of gonadal mosaicism, it is higher than in the general population.

## Background

Aniridia (OMIM #106210 [1]) is a Mendelian autosomal dominant complex congenital developmental disorder that can affect all eye structures as well as central nervous system, the endocrine system, and other systems and organs [2, 3]. Congenital aniridia (AN) is caused by heterozygous mutations in the *PAX6* gene (OMIM *607108; 11p13) or 11p13 deletions. Small intragenic mutations have been found in about 50–70% of aniridia cases, and large chromosome rearrangements are found in about 30–40%. Deletions can involve one or more exons, the entire *PAX6* gene and several neighboring genes, as well as 3′-*cis*-regulatory region, with some expanded deletions capture area including genes *PAX6* and *WT1* [4, 5]. This last type of 11p13 chromosome region deletions is known as WAGR-region deletions. They occur in 7–12% of all cases and in approximately 30% of sporadic cases of congenital aniridia, and they lead to WAGR syndrome (OMIM #194072, **W**ilms’ tumor, **A**niridia, **G**enitourinary anomalies, and development **R**etardation) [6].

The median age of development of WAGR-associated kidney tumor is about 2 years old. In newborns, WAGR syndrome can be manifested by a single sign – aniridia or hypoplasia of the iris. De novo chromosome deletion of the “WAGR-region” determined in a patient with sporadic aniridia increases the risk of Wilms’ tumor dramatically. Wilms’ tumor develops in 40–60% of patients with “WAGR-region” deletions [7, 8].

Molecular diagnostics is therefore needed to be taken as soon as possible. In addition, some chromosome rearrangements involving 11p13 are known to be more complex than simple interstitial deletions [9]. If healthy parents have balanced chromosome rearrangements, the risk of having another affected child increases significantly. In this scenario deletions are formed because of inversions or translocations. Such deletions constitute as many as 10–17% of all detected 11p13 chromosome rearrangements [9, 10].

Here we present comprehensive DNA diagnosis of a sporadic case of congenital aniridia with hydrocephalus and early CNS damage associated with pericentric chromosome 11 inversion with an approximately 1-Mb deletion at the breakpoint in the 11p13 region. The applied approaches in this and other cases of complex 11p13 chromosome rearrangements help to make early differential diagnosis of WAGR syndrome and to determine the risk having the parents having another affected child.

## Case presentation

**Fig. 1:**
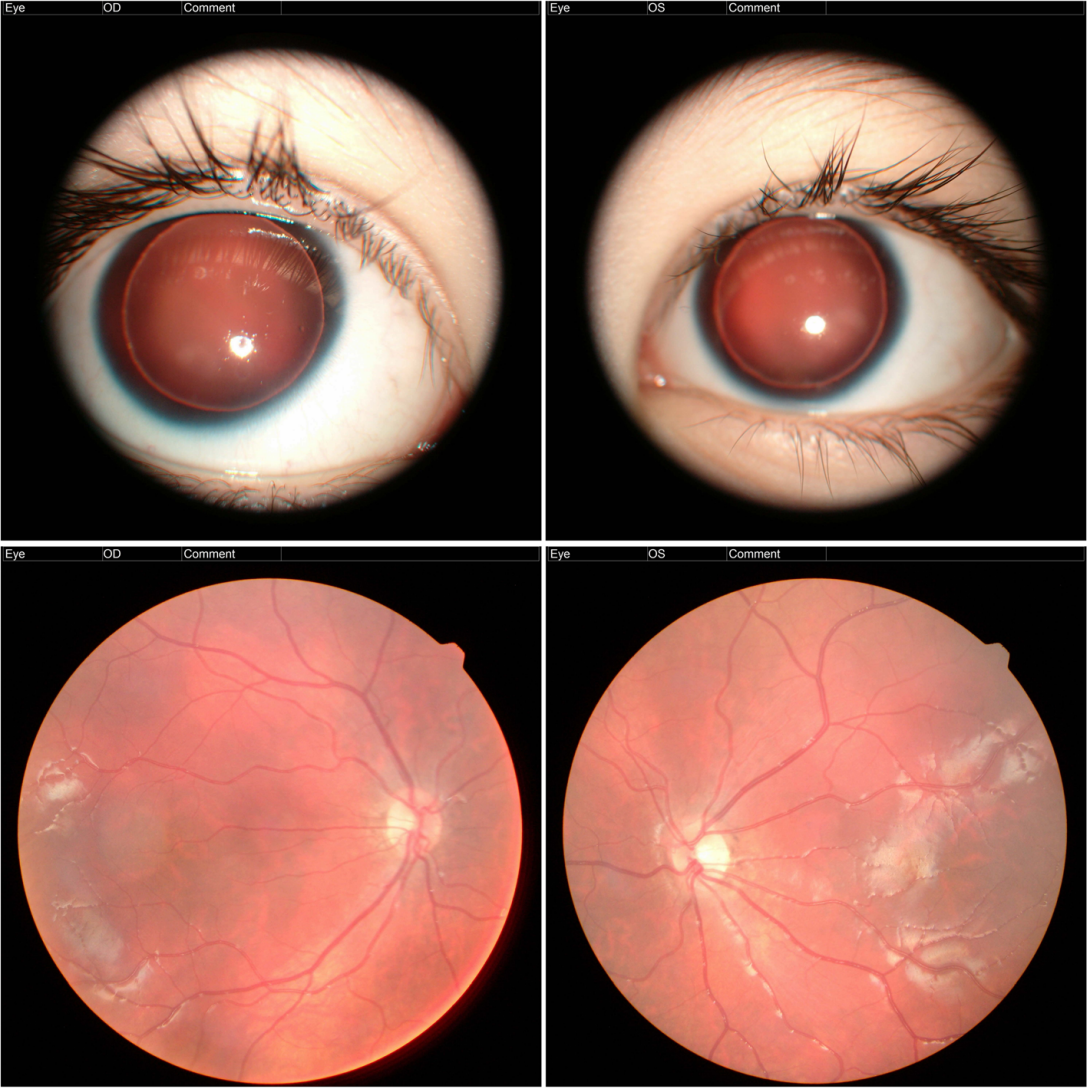
Slit lamp view of left and right eye (top); Single fundus photograph of left and right eye (bottom)

The patient is a 2-year-old boy, the second child in the family. His elder brother and parents are healthy. Ophthalmological, neurological, and ultrasonographic examination of the patient showed complete aniridia, cataract, optic disc hypoplasia and partial atrophy of the optic nerves, foveal hypoplasia, nystagmus, hypotalamia (shallow anterior chamber), high hypermetropia, and strabismus; (Fig. 1), as well as early organic CNS damage, hydrocephalus, brain vascular plexus cysts, developmental delay, myotonic syndrome, pes valgus, ataxia, and emotional lability. In addition, short stature, gallbladder dysfunction, reactive pancreatitis, iodine deficiency, anemia, celiac disease, atopic dermatitis, open oval window, and umbilical hernia were observed.

**Fig. 2:**
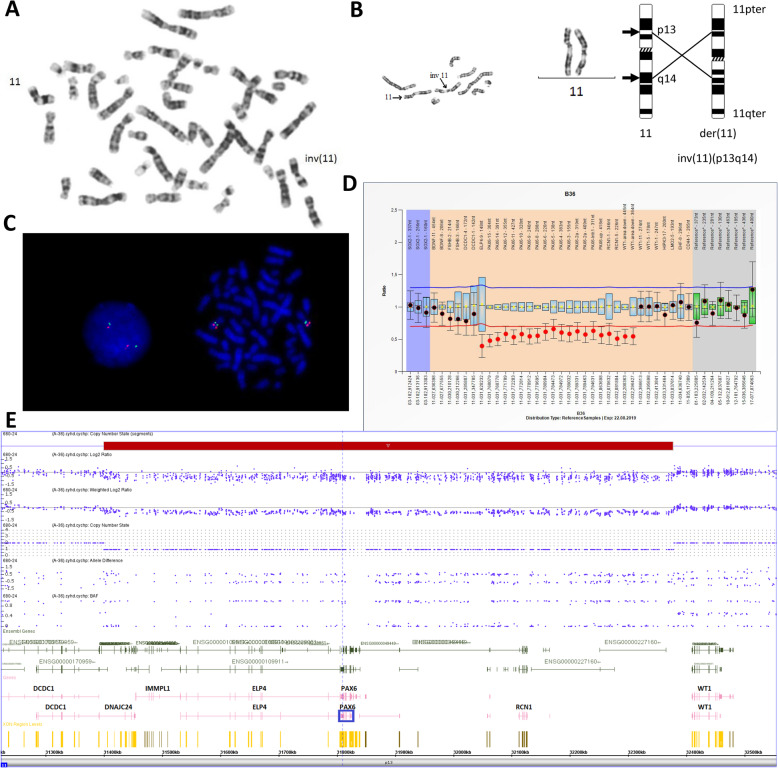
**a**. Patient’s karyotype. **b**. Detailed view on derivative chromosome 11 and ideogram view. **c**. FISH analysis on *WT1* locus. **d**. MLPA analysis for the 11p13 region. **e**. CMA analysis of the 11p13 region

Initial cytogenetic study revealed the normal karyotype 46,XY and pericentric inversion of chromosome 11, inv(11)(p13q14) (Fig. 2 a, b). The best resolution of the karyotyping was about 10 Mb. Inversion was not identified in the healthy parents of the proband with normal karyotype (data not shown) thus it was assumed to occur de novo. Such an inversion could lead to the patient’s phenotype with aniridia in two ways: either through the so-called position effect earlier described for AN [11], or through the loss of genomic material at the rearrangement break points. In the latter case the refinement of the deletion boundaries is crucial due to the vicinity of the *WT1* gene.

To determine a possible chromosome disbalance in the 11p13 region, multiplex ligase-dependent probe amplification (MLPA) analysis was applied using the SALSA MLPA of P219-B2 PAX6 probes (MRC-Holland, Amsterdam, the Netherlands). The MLPA analysis showed a deletion stretched for at least 668 kb in 11p13 region (hg19::chr11:g.(30255690_31671656)_(32339851_32410037)del) and removed loci of the *ELP4*, *PAX6*, and *RCN1* genes, and it did not affect the *WT1* gene (Fig. 2d). This was confirmed by normal fluorescence in situ hybridization (FISH) pattern with a specific probe for the *WT1* gene locus (FA0275, Abnova, Taiwan) and 11p region (CEN11p, FC0096, Abnova). (Fig. 2c). Further high-resolution chromosomal microarray analysis (CMA) with CytoScan HD array (2.67 M probes, ThermoFisher Scientific, MA USA) specified the deletion region to be 977.065 kb (arr [GRCh37] 11p13(31400114_32377179)×1) (Fig. 2e). The CMA revealed no meaningful disbalance of more than 10 kb in the 11q14 region at the other side of the inversion.

## Discussion and conclusions

Thus, we have established molecular-genetic confirmation of the congenital aniridia diagnosis of the proband. The deletion and inversion identified in the proband was not found in his parents, and the risk of the birth of another child with aniridia in the family is slightly higher than in the general population (0.001%) considering the possibility of gonadal mosaicism and, thus, requires the prenatal diagnosis if needed. For the patient, the risk of development of Wilms’ tumor is similar to the risk in the general population (0.01%) [12].

Balanced chromosomal rearrangement is a possible mechanism for the formation of the 11p13 deletion, because such chromosomal deletion is a very frequent cause of AN (determined in one third of cases) [6]. Nevertheless, there are only a few descriptions of chromosome 11 inversions in the literature [13].

Our proband, like some other aniridic patients, exhibits complex congenital eye malformation accompanied by the involvement of the central nervous system and other systems. About 10% of patients with aniridia have been described to have systemic features, such as: myotonic syndrome, developmental delay, sleep disturbance, and emotional lability, and less often (< 10%) seizures, epilepsy, and other neurological complications, as well as endocrine dysfunction of the pituitary gland, thyroid, and pancreas, malformations of the kidneys and urinary and gall bladders, immune diseases, and connective tissue and skeletal defects [14].

All these observations point to syndromic character of the hereditary disorder of congenital aniridia. For this reason, the term “*PAX6* syndrome” has recently come into use, as it is more appropriate than just “congenital aniridia” [6, 15]. Thus, our patient has severe syndromic character of congenital aniridia. On one hand, the severity of aniridia phenotype including systemic complications is not determined solely by the type of mutation, and it can be different for all types of mutations, including large deletions of the 11p13 region [16]. On the other hand, while a causative mutation is chromosomal deletion, decrease of function of several genes captured within the deleted region could be responsible for systemic complications observed in patients. The question of the contribution of *PAX6* neighboring genes in the 11p13 region to the manifestation of congenital aniridia remains open. Additionally, when chromosome rearrangements are complex, spatial genomic disorganization could worsen the severity of systemic complications [17, 18]. It was recently shown that chromosomal aberrations might not lead to disbalance due to gain or loss of genetic material but rather to the disruption of topologically associating domains (TADs), when a large structural variant might disrupt a CTCF-binding boundary, leading to changes in regulation of unaffected genes [19].

This reported case of congenital aniridia in our patient with a pericentric inversion associated with a 977.065-kb deletion at the breakpoint in the 11p13 region demonstrates the need for thorough genetic analyses in all cases of congenital aniridia caused by chromosomal disbalance in 11p13 for both probands and their parents.

## Data Availability

The datasets used and/or analyzed during this study are available from the corresponding authors on reasonable request.

## Abbreviations

AN: Congenital aniridia
CMA: Chromosomal microarray analysis
CNS: Central nervous system
FISH: Fluorescence in situ hybridization
MLPA: Multiplex ligase-dependent probe amplification
OMIM: Online Mendelian Inheritance in Man
TADs: Topologically associating domains
WAGR syndrome: Wilms’ tumor, Aniridia, Genitourinary anomalies, and development Retardation
